# Impacts of COVID-19 on sexual risk behaviors, safe injection practices, and access to HIV services among key populations in Zambia: Findings from a rapid qualitative formative assessment

**DOI:** 10.1371/journal.pone.0289007

**Published:** 2023-08-01

**Authors:** Lauren E. Parmley, Tepa Nkumbula, Lophina Chilukutu, Lazarus Chelu, Chipili Mulemfwe, Brave Hanunka, John Mwale, Joyce Neal, Ray Handema, Prisca Kasonde, Kennedy Mutale, Henry M. Sakala, Maria Lahuerta

**Affiliations:** 1 ICAP at Columbia University, New York, NY, United States of America; 2 ICAP at Columbia University, Lusaka, Zambia; 3 Division of Global HIV & TB, Center for Global Health, U.S. Centers for Disease Control and Prevention, Lusaka, Zambia; 4 National HIV/AIDS/STI/TB Council, Lusaka, Zambia; 5 Division of Global HIV & TB, Center for Global Health, U.S. Centers for Disease Control and Prevention, Atlanta, GA, United States of America; 6 Tropical Diseases Research Centre, Ndola, Zambia; 7 Key Populations Consortium, Lusaka, Zambia; 8 Department of Epidemiology, Mailman School of Public Health, Columbia University, New York, NY, United States of America; HIV/STI Surveillance Research Center and WHO Collaborating Center for HIV Surveillance, Institute for Future Studies in Health, Kerman University of Medical Sciences, ISLAMIC REPUBLIC OF IRAN

## Abstract

**Background:**

Despite achievements in the HIV response, social and structural barriers impede access to HIV services for key populations (KP) including men who have sex with men (MSM), transgender women (TGW), and people who inject drugs (PWID). This may be worsened by the COVID-19 pandemic or future pandemic threats. We explored the impact of COVID-19 on HIV services and sexual and substance use behaviors among MSM/TGW and PWID in Zambia as part of a formative assessment for two biobehavioral surveys.

**Methods:**

From November-December 2020, 3 focus groups and 15 in-depth interviews (IDIs) with KP were conducted in Lusaka, Livingstone, Ndola, Solwezi, and Kitwe, Zambia. Overall, 45 PWID and 60 MSM/TGW participated in IDIs and 70 PWID and 89 MSM/TGW participated in focus groups. Qualitative data were analyzed using framework matrices according to deductive themes outlined in interview guides.

**Results:**

KP reported barriers to HIV testing and HIV treatment due to COVID-19-related disruptions and fear of SARS-CoV-2 exposure at the health facility. MSM/TGW participants reported limited supply of condoms and lubricants at health facilities; limited access to condoms led to increased engagements in condomless sex. Restrictions in movement and closure of meet-up spots due to COVID-19 impeded opportunities to meet sex partners for MSM/TGW and clients for those who sold sex. COVID-19 restrictions led to unemployment and loss of income as well as to shortages and increased price of drugs, needles, and syringes for PWID. Due to COVID-19 economic effects, PWID reported increased needle-sharing and re-use of needles.

**Conclusions:**

Participants experienced barriers accessing HIV services due to COVID-19 and PWID attributed unsafe needle use and sharing to loss of income and lack of affordable needles during pandemic-related restrictions. To maintain gains in the HIV response in this context, strengthening harm reduction strategies and improvements in access to HIV services are necessary.

## Introduction

Social and structural vulnerabilities, including stigma and discrimination, increase HIV acquisition risk and impede access to HIV services for key populations (KP) including sex workers, gay, bisexual, and other men who have sex with men (MSM), transgender individuals such as transgender women (TGW), and people who inject drugs (PWID) [1–3]. In sub-Saharan Africa, half of all new HIV infections occur among KP and their sexual partners [4]. Risk of acquiring HIV is 28 times higher among MSM than adult men, 14 times higher among TGW than adult women, and 35 times higher among PWID than people who do not inject drugs [4].

As COVID-19 has reinforced health inequities among marginalized groups, social and structural barriers affecting HIV risk and access to services for KP could be worsened by the COVID-19 pandemic or other emerging pandemic threats. Disruptions in health commodities and routine health services for HIV, as have been documented during periods of COVID-19 lockdown across Africa and Asia [5], may disproportionately affect KP given their high HIV burden. While there is no clear evidence that people living with HIV have higher risk of SARS-CoV-2 infection, people living with HIV may be more likely to suffer severe illness from COVID-19 and have higher in-hospital mortality than people not living with HIV based on global hospitalization data across 38 countries [6]; given their HIV burden, KP may disproportionately experience COVID-19 complications and adverse outcomes relative to the general population.

In Zambia, available data on population-level HIV prevalence and population size estimates of KP are limited and there are few legal protections for KP. Consensual same-sex conduct is criminalized [7], and punitive drug and drug equipment laws pose challenges to harm reduction service delivery [8,9]. Moreover, legal structures and cultural contexts contribute to stigma and discrimination and increase risk of violence and abuse for KP [10].

From March-September 2020, in response to the COVID-19 pandemic, Zambia instituted a partial lockdown and closed establishments including sports clubs, nightclubs, bars, casinos, and restaurants, as well as implemented social distancing measures and restrictions. Government mitigation strategies or mandates in response to COVID-19 or future pandemic threats, like those implemented in Zambia, have the potential for further stigmatization of KP, including police targeting and loss of income, and may be difficult to adhere to for KP, particularly those who are homeless or those with overcrowded living conditions [11–13].

We qualitatively explored the impact of COVID-19 and government restrictions to manage COVID-19 on HIV services and sexual and substance use behaviors among MSM/TGW and PWID as part of a formative assessment to inform two upcoming biobehavioral surveys (BBS), the first respondent-driven sampling (RDS) surveys to be conducted with these groups, in Zambia.

## Materials and methods

Data were collected from November-December 2020, two months after the partial lockdown was lifted. Survey towns included Lusaka (MSM/TGW, PWID), Livingstone (MSM/TGW, PWID), Ndola (PWID), Solwezi (MSM/TGW), and Kitwe (MSM/TGW), Zambia. MSM/TGW survey towns were selected where prior MSM population size estimates were highest according to an unpublished study [14]. PWID survey towns were selected to include three highly populous cities in Zambia, which have a high number of people who use drugs according to population size estimates from the same unpublished study [14]. In each survey town, 3 focus groups discussions (FGDs) and 15 in-depth interviews (IDIs) with each KP were conducted. Overall, 45 PWID and 60 MSM/TGW participated in IDIs and 70 PWID and 89 MSM/TGW participated in FGDs.

Participants were recruited using purposive sampling techniques and through snowball sampling to ensure diversity across demographic characteristics. Recruitment efforts were supported by KP community mobilizers and KP civil society organizations. MSM/TGW were eligible to participate if they were assigned male at birth, self-reported anal or oral sex with a man in the past 6 months, were at least 16 years of age, lived in the survey town for at least 3 months, spoke English or any other designated local language, and were able to provide informed consent. PWID were eligible to participate if they self-reported drug injection for non-medical purposes in the past month, were at least 16 years of age, lived in the survey town for at least 3 months, spoke English or any other designated local language, and were able to provide informed consent.

Where possible, FGDs for each KP group were stratified by participant characteristics (i.e., gender and age) to encourage participants to freely share ideas and perceptions. After a brief introduction of the upcoming BBS, interviewers obtained verbal informed consent from each eligible participant. As part of the consent process, potential benefits of participating in the formative assessment were discussed, including potential to ensure KP perspectives and experiences informed BBS methods and KP service delivery. The legal age of consent in Zambia is 16 years hence parental/guardian consent was not sought nor required for participation. However, referrals to local resources were provided to participants less than 18 years who reported sex work or who reported being a victim of violence or trafficking. FGD participants were instructed not to use their name, the name of other participants, or other KP that could suffer negative consequences if identified through FGDs. IDIs and FGDs were conducted by two trained interviewers (a moderator and a note taker) with the aid of semi-structured IDI/FGDs guides at separate, private locations for MSM/TGW and PWID. Verbal informed consent and IDIs/FGDs were conducted in the participant’s preferred language (Chinyanja, Chitonga, Cibemba, Kikaonde, Silozi, or English). All participants were reimbursed Zambian kwacha equivalent of $13 to cover transportation costs and time. Refreshments including a bottle of water, a snack, and/or soft drink were offered and provided to participants.

IDI and FGD guides were adapted from formative assessment guides used with MSM and others assigned male who have sex with men in neighboring Zimbabwe [15]. During IDIs/FGDs, interviewers generated memos as the discussion unfolded to help formulate follow-up questions and probes. Interviewers documented their impressions about the sessions, its main themes and participant quotes using a deductive interview notes template according to IDI/FGD guide categories. Following IDIs/FGDs, teams also held debrief sessions to discuss emerging themes. Data from the deductive notes template were subsequently populated into an Excel matrix; categories were analyzed separately for each KP group, survey town, and data collection method by two team members [16]. For each KP, across case and within case comparisons were made and additional comparisons by age and gender were reviewed for select categories [17]. Themes presented reached data saturation—the point at which further inquiry did not yield any additional information [18,19].

Ethical and administrative approvals were received from the Columbia University Institutional Review Board, the Tropical Diseases Research Centre Ethics Review Committee, and the Zambia National Health Research Authority. The protocol was also reviewed in accordance with the United States Centers for Disease Control and Prevention (CDC) human research protection procedures and was determined to be research, but CDC investigators did not interact with human subjects or have access to identifiable data or specimens for research purposes. Additional information regarding the ethical, cultural, and scientific considerations specific to inclusivity in global research is included as (S1 Questionnaire).

## Results

### Impacts of COVID-19 among MSM and TGW

Duration of sessions ranged from 55–150 minutes for IDIs and 90–230 minutes for FGDs. MSM/TGW IDI participants ranged in age from 16–55 years (Table 1). More than a quarter of participants identified as TGW (28%). Most had received a secondary school education (48%) and worked in the informal sector (57%). FGDs included between six to nine participants. Nine FGDs consisted of participants 25 years or older (75%) and three FGDs consisted of participants less than 26 years (25%).

**Table 1:** Demographic characteristics among MSM/TGW IDI participants, Zambia, 2020.

Demographic characteristic	Lusaka (n = 15, 25%)	Livingstone (n = 15, 25%)	Solwezi (n = 15, 25%)	Kitwe (n = 15, 25%)	Total (n = 60, 100%)
Median age (range)	24 (18–32)	28 (20–55)	26 (21–46)	29 (16–45)	26 (16–55)
Key population MSM TGW*	8 (53%) 7 (47%)	10 (67%) 5 (33%)	14 (93%) 1 (7%)	11 (73%) 4 (27%)	43 (72%) 17 (28%)
Education Primary Secondary Tertiary/vocational	4 (27%) 9 (60%) 2 (13%)	1 (7%) 7 (47%) 7 (47%)	0 (0%) 7 (47%) 8 (53%)	2 (13%) 6 (40%) 7 (47%)	7 (12%) 29 (48%) 24 (40%)
Employment Formal Informal Student	3 (20%) 6 (40%) 6 (40%)	6 (40%) 7 (47%) 2 (13%)	5 (33%) 9 (60%) 1 (7%)	3 (20%) 12 (80%) 0 (0%)	17 (28%) 34 (57%) 9 (15%)
Marital status Single/never married Married to one or more men Married to one or more women Cohabitating Separated/divorced	14 (93%) 1 (7%) 0 (0%) 0 (0%) 0 (0%)	10 (67%) 2 (13%) 0 (0%) 2 (13%) 1 (7%)	12 (80%) 1 (7%) 1 (7%) 0 (0%) 1 (7%)	9 (60%) 3 (20%) 1 (7%) 2 (13%) 0 (0%)	45 (75%) 7 (12%) 2 (3%) 4 (7%) 2 (3%)
Religion Christian Other	14 (93%) 1 (7%)	11 (73%) 4 (27%)	15 (100%) 0 (0%)	15 (100%) 0 (0%)	55 (92%) 5 (8%)

*Includes participants who specified their current gender as female or transwoman.

Six primary themes related to the impacts of COVID-19 on MSM and TGW emerged from IDIs and FGDs: economic impact, mental health, HIV services, stigma and discrimination, sexual behavior, and, relevant to the objectives of the formative assessment, willingness to participate in the upcoming BBS. Illustrative quotes for themes and sub-themes are provided in Table 2.

**Table 2:** Themes, sub-themes, and illustrative quotes on the impacts of the COVID-19 pandemic among MSM and TGW, Zambia, 2020.

Sub-theme	Illustrative quote(s)
**Primary theme 1: COVID-19 economic impacts**	
Loss of formal employment	*“[I] was laid off from work due to the lockdown induced by COVID-19*.*”–Kitwe IDI*, *MSM 25 years* *“Key populations work in bars and their jobs were terminated and the places where MSM used to hangout were closed as well*. *Money became very difficult to find at that time*.*”–Lusaka IDI*, *MSM 26 years*
Loss of income through self-employment	*“The lockdown brought our business down*. *For me who runs a saloon*, *weddings*, *kitchen parties*, *and just parties bring money for me*. *With the lockdown*, *all these were not allowed*. *Meaning paying for my shop and the house I rent was now an issue*.*”–Solwezi IDI*, *TGW 21 years* *“COVID-19 has affected me and my business a lot*. *I run an electronic shop so from the time this came*, *my movements to get more products were restricted and this led to loss of business*.*”–Solwezi IDI*, *MSM 24 years*
Loss of income through sex work	*“Business became very slow as there were no clients willing to meet up during the pandemic*.*”–Lusaka FGD*, *30–42 years* *“It brought about a lot of suffering because shops and bars were closed*. *So*, *chances to meet with would be clients were reduced*.*”–Kitwe IDI*, *MSM 33 years*
**Primary theme 2: COVID-19 impacts on mental health**	
Anxiety due to isolation/quarantine	*“[I] used to feel very lonely and alone though I was around family”–Kitwe IDI*, *TGW 16 years* *“It [COVID-19] did stress me because there was no interaction and that caused me stress and anxiety”–Livingstone IDI*, *MSM 28 years* *“Feeling of being caged because of movement restrictions”–Kitwe FGD*, *25–40 years*
Stress due to economic impacts of COVID-19	*“I always wondered about how to meet my basic needs because business was affected badly”–Solwezi IDI*, *MSM 34 years* *“I had a friend who was affected mentally due to the lockdown as it prevented his clients from Botswana [from] coming over to pay for sex making him broke*.*”–Livingstone IDI*, *MSM 27 years* *“[I] was depressed because [I] didn’t have money and was anxious about the future*.*”—Lusaka IDI*, *TGW 32 years*
Fear of contracting SARS-CoV-2	*“I was affected mentally and became stressed for fear of contracting COVID-19”–Livingstone IDI*, *TGW 21 years* *“[I] was depressed…scared even of white clients this is so because of a myth that white people are prone to the virus [more] than black people and this caused [me] to avoid all [of my] white clients*.*”–Lusaka IDI*, *MSM 26 years*
**Primary theme 3: COVID-19 impacts on HIV services**	
Prioritization of COVID-19 patients at health facility; turned away if non-COVID-19 patient	*“It was difficult to access HIV services because those who distributed condoms*, *lubricants and just test kits for HIV stopped and all the focus was on COVID*.*”–Solwezi FGD*, *<25 years* *“Most people were being told to go back home because the clinics wanted a small number of people to attend to and only people with serious cases were allowed to access services at the clinics*.*”–Lusaka FGD*, *25–31 years*
Operational changes at health facility such as reduced hours and restrictions on the number of patients indoors	*“We were not able to access services because hours were reduced*, *and movements were restricted*.*”–Solwezi IDI*, *MSM 21 years* *“It was hard to access services*, *during that time we could not be [in] crowds*.*”–Solwezi IDI*, *MSM 28 years*
Mandatory screening or testing for COVID-19 prior to accessing HIV services; mandatory mask requirements	*“Before they could attend to you*, *they could first screen you for COVID-19*, *so it used to consume a lot of time for you to be attended to*.*“–Kitwe IDI*, *MSM 31 years* *“[We] are chased away from the clinic if not wearing a mask”–Kitwe FGD*, *25–40 years* *“There were a lot of restrictions like masking up and the process of COVID screening was time consuming”–Kitwe IDI*, *MSM 36 years*
Longer wait time at health facility	*“To do an HIV test was hard because only a few people could be attended to at a time*, *usually I could feel lazy to wait and would eventually just go home*. *In addition to this*, *there was less working hours due to the COVID-19 pandemic especially [within] the ART department*.*”–Solwezi IDI*, *MSM 26 years*
Limited supply and access to condoms, lubricants, and HIV prevention due to lockdown and border closures	*“It became so difficult to get condoms at the clinics”–Lusaka FGD*, *25–31 years* *“My practicing of safe sex safe was affected because there were no condoms as well as lubricants*.*”–Solwezi IDI*, *MSM 22 years* *“There was no access to lubricants as their importation became difficult because of the lockdown*.*”–Kitwe IDI*, *MSM 25 years*
Avoidance of HIV care seeking due to fear of SARS-CoV-2	*“Can’t get tested because you are scared of getting corona from the hospital”–Kitwe IDI*, *MSM 17 years* *“Because of the restrictions in the movements*, *we were failing to go and access HIV services*. *For me I was very much afraid of hospitals or clinics I thought they could be the source of COVID-19 since patients are taken there”–Solwezi IDI*, *MSM 23 years*
Drug stockouts including stock out of antiretrovirals	*“The effect was big on the part of clients especially those who used to get TLD*, *it ran out and they started giving them AVONZA*, *drugs which they had been using before*.*”–Kitwe IDI*, *MSM 33 years* *“No proper care [at the health facility]*, *at times [we were] told that drugs are out of stock”–Kitwe IDI*, *MSM 29 years*
Adaptions to HIV service delivery, including HIV self-testing, mobile clinics, appointment requirements	*“One had to set an appointment*, *but before the pandemic one could just walk in*.*”–Lusaka IDI*, *TGW 32 years* *“These days we have self-testing kits in our homes*.*”–Solwezi IDI*, *MSM 25 years* *“[Name of organization] formed a WhatsApp group that was meant for collecting condoms at specific period[s] of time to avoid overcrowding at the organization…health workers from the [clinic name] used to pass through communities using a mobile health vehicle and administered condoms to people*.*”–Lusaka FGD*, *25–31 years*
Non-COVID-19-related barriers to care; stigma experienced at health facilities	*“Once you open about your situation and they realize it is [an] MSM health-related issue they refuse to treat us; they tell us to go back and get it treated wherever we got it from or tell us to go to [a] private facility*.*”–Solwezi IDI*, *MSM*, *34 years* *“MSM and TGW are not treated well at government facilities that is why I don’t even go there*. *We are not respected like anyone else…When we go there*, *they always want to change who we are because they feel it is something we can change at any time*. *They feel any sexual disease that we have is our fault*.* *.* *. *We are not given the privacy that we desire*.*”–Solwezi IDI*, *TGW*, *21 years*
**Primary theme 4: COVID-19 impacts on sexual behavior and condom use**	
Increased sexual behavior due to boredom during lockdown	*“A lot of sex was done during the lockdown period due to boredom*, *starvation and people staying indoors for a long time with less or nothing to do*. *The use of condom was somehow forgotten during lockdown”–Lusaka IDI*, *MSM 20 years* *“Sex hookups increased as MSM started using pimps to have sex with men*. *This was mostly through other MSM*, *and it encouraged the sharing of partners as sex became a social thing because there was nothing to do*.*”–Kitwe FGD*, *25–40 years*
Reduced sexual behavior due to fear of SARS-CoV-2 exposure	*“We cannot risk our lives by being involved in sexual behaviors that would expose us to the risk of contracting the virus that cause COVID”—Solwezi FGD*, *25–41 years* *“It has affected our sex life because we can no longer have sex the way we used to have it*. *With the social distance that was being encouraged*, *people were afraid to engage into that”–Solwezi IDI*, *MSM 21 years*
Reduced sexual behavior due to restrictions in movement, border closure, and closure of locations to have sex (especially in Livingstone)	*“We rarely met*, *interactions reduced*, *even places where to meet were closed”–Kitwe IDI*, *MSM 32 years* *“COVID has affected our sexual behaviors in that our partners especially those that come from outside the country cannot travel to come see us*. *Number of sexual partners has gone down compared before COVID”–Livingstone IDI*, *MSM 28 years* *“We’re also affected as we couldn’t meet up with partners*. *For example*, *my partner lives in Vicfalls Town*, *Zimbabwe and so we would only talk on the phone*.*”–Livingstone*, *MSM 28 years*
Limited access to condoms led to engagements in condomless sex	*“We are forced to have unprotected sex*. *No condoms and lubricants during Corona”–Kitwe IDI*, *MSM 17 years* *“During this time*, *a lot of MSM had a lot of unprotected sex and this scared me”—Lusaka IDI*, *MSM 26 years* *“My practicing of safe sex safe was affected because there were no condoms as well as lubricants*.*”–Solwezi IDI*, *MSM 22 years*
Reduced number of partners; Unable to meet new partners due to lockdown restrictions and border closures (particularly in Livingstone), and fear of SARS-CoV-2	*“Most places have been shut down making it difficult to find or meet new sexual partners”–Livingstone IDI*, *MSM 28 years* *“COVID affected finding new sexual partners*. *Initially pre-COVID*, *I had 10 sexual partners*, *but the COVID era reduced that to zero*.*”–Livingstone IDI*, *TGW 21 years* *“I couldn’t find new partners*, *operating hours for bars*, *clubs everything to do with social were reduced*. *The number of partners really reduced”–Solwezi IDI*, *TGW 21 years*
Sex workers unable to meet clients due to lockdown restrictions, border closures, and fear of SARS-CoV-2; loss of income through sex work	*“It was hard under COVID…the numbers reduced especially for us who do sex work*. *Clients that pay well got scarce*.*”–Livingstone IDI*, *MSM 27 years* *“Please talk of us who sell sex for money*, *there was no business*. *A lot of whites were not coming out of the mines [where they work]*. *They were under lock down… Accessing lubricants and condoms was an issue*.*”–Solwezi IDI*, *TGW 21 years* *“It brought about a lot of suffering because shops and bars were closed*. *So*, *chances to meet with would be clients were reduced*.*”–Kitwe IDI*, *MSM 33 years*
Increased engagement to meet new partners/clients via social media platforms	*“No new hook-ups except for social media apps such [as] Facebook [or] through other friends”–IDI Kitwe*, *MSM 17 years* *“MSM are now finding new sexual partners through social media platforms such as Facebook*, *Grindr and WhatsApp”–Kitwe FGD*, *25–40 years* *“We are using apps like Grindr to meet new partners and make new friends*.*”–Livingstone IDI*, *MSM 28 years* *“It wasn’t hard to find new sexual partners because this was possible through social media*.*”–Solwezi IDI*, *MSM 22 years*
Meet-ups in homes or other private locations due to closure of usual meet-up spots	*“We could not meet in the usual places we used to meet*. *So sometimes we went to their [partner’s] homes”–Solwezi IDI*, *MSM 46 years* *“We could sometimes go to their [partner’s] homes after contacting them on the phone”–Kitwe IDI*, *MSM 32 years*
**Primary theme 5: COVID-19 impacts on stigma and discrimination**	
Reduction in public stigma/discrimination due to restrictions in movement, less socialization, bar closure, etc. where violent acts occurred	*“Because of the restrictions in movements*, *stigma and discrimination reduced because MSM could meet very few people in bars and streets”–Kitwe FGD*, *25–40 years* *“During lockdown*, *stigma was on the lower side than in normal times*.*”–Livingstone IDI*, *MSM 28 years* *“No stigma experienced because I was in a lock down home alone*.*”—Solwezi IDI*, *MSM 25 years*
Similar levels of stigma perpetuated by family due to government lockdown	*“There has been less stigma during COVID because of less contact with others though we still face stigma in homes*.*”–Livingstone IDI*, *TGW 25 years* *“The stigma was still the same especially from family members who suspect that [I] am MSM*.*”–Livingstone IDI*, *TGW 20 years*
**Primary theme 6: Willingness to participate in upcoming BBS in the context of COVID-19**	
COVID-19 is unlikely to impede participation as COVID-19 is the “new normal”	*“No*, *it [COVID-19] wouldn’t hinder us from participating*. *It’s the new normal*. *We would gladly participate*.*”–Livingstone IDI*, *TGW 30 years* *“P04*: *The survey you’re bringing is very good and I don’t think COVID-19 will discourage any MSM from participating*.* *.* *.*P05*: *Don’t worry people will come*, *this is the new normal now*.*”–Solwezi FGD*, *25–41 years* *“Am willing to take part and our participation with peers is not affected as we have adopted the new normal way of carrying ourselves like masking up*, *handwashing*, *and indeed social distancing*.*”–Livingstone IDI*, *TGW 21 years*
A worsening epidemic and/or increased government containment measures may impact participation	*“Interested to participate*, *but if COVID continues*, *restrictions will be there*, *so people will fail to attend*.*”–Kitwe IDI*, *MSM 36 years* *“They [MSM] may be scared that their participation would expose them to contracting the Corona Virus”–Kitwe IDI*, *MSM 17 years*
Transmission mitigation strategies such as distribution of masks and sanitizer and implementation of social distancing may encourage participation	*“There are precautions that have been outlined to be adhered to in order to protect ourselves*. *So just provide the facemask*, *sanitizer and maintain social distance*. *When you do this people will come*.*”–Solwezi FGD*, *25–41 years* *“To make participants more comfortable about coming to the survey during this time of COVID-19*, *this is going to be achieved through observing the COVID-19 rules*, *it is the new normal after all*.*”–Solwezi IDI*, *MSM 22 years* *“As long as we can follow the COVID-19 health guidelines then everything will be okay*.*”–Solwezi IDI*, *MSM 26 years*

Across towns, MSM and TGW described severe economic impacts resulting from the COVID-19 pandemic, including loss of formal employment and loss of income for those who were self-employed and/or working as sex workers. Movement restrictions and the temporary closure of public places such as bars and restaurants impeded business for participants with formal employment and limited opportunities for MSM and TGW sex workers to meet clients.

COVID-19 contributed to adverse mental health outcomes. MSM and TGW in all cities and across data collection methods reported experiencing anxiety due to isolation and/or quarantine during the pandemic. Participants reported feeling isolated from family and friends both physically and emotionally. The economic impacts of COVID-19 further exacerbated anxieties for participants. Participants reported experiences of depression and anxiety about meeting financial commitments and their ability to afford necessities. Fear of SARS-CoV-2 exposure was reported as an additional anxiety for MSM and TGW during the pandemic; participants reported this fear motivated them to change behavior, including remaining in their homes, avoiding healthcare settings, and influencing their sexual behaviors.

All participants across data collection methods reported impacts of COVID-19 on HIV service delivery. Participants reported limited supply of condoms and lubricants at health facilities and barriers to HIV testing due to COVID-19-related disruptions and operational changes; prioritization of COVID-19 patients at health facilities and being turned away when presenting with non-COVID-19 health issues; avoidance of HIV care-seeking due to fear of SARS-CoV-2 exposure; and antiretroviral therapy (ART) stockouts. Limited access to condoms also led to increased engagements in condomless sex. While participants described adverse impacts of COVID-19 on access to HIV services, they also described its benefits, including the implementation of innovations and adaptions to HIV service delivery in response to the pandemic such as HIV self-testing, mobile clinics, and appointment reminders.

In addition to COVID-19-related barriers, MSM/TGW reported experiencing stigma and discrimination and poor-quality health services within public health facilities irrespective of COVID-19 due to non-MSM/TGW friendly staff across cities. MSM/TGW participants reported that negative attitudes from healthcare workers and lack of confidentiality deterred them from accessing services at public facilities. Healthcare workers were reported to routinely stigmatize and mistreat patients who identified as MSM/TGW. As a result, many MSM/TGW avoided visiting public health facilities and preferred accessing health services at private clinics and non-governmental organizations before and during COVID-19. Those who accessed services at public facilities reported feeling comfortable interacting with staff who were a part of the KP community; many reported not disclosing anal sex behaviors with staff to prevent stigmatization and/or discrimination.

Restrictions in movement and closure of usual meet-up spots due to COVID-19 impeded opportunities to meet sex partners and clients for MSM/TGW who sold sex. In response, participants used social media to meet sexual partners/clients and met in homes/private locations. While some participants reported an increase in sexual behavior due to boredom, most reported reduced sexual activities due to fear of SARS-CoV-2 exposure, movement restrictions, and closure of lodges/bars.

Participants reported that restrictions in movement and limited socialization in public areas where stigma, discrimination, and violence ‘typically’ occur resulted in reduced experiences of stigma and discrimination in public settings during the pandemic. Yet, mobility restrictions had no impact on reducing experiences of stigma from family and/or household members as MSM and TGW spent longer periods of time in the home because of the lockdown.

In response to prompts around interest and willingness to participate in the upcoming BBS in the context of COVID-19, most participants reported that COVID-19 was unlikely to impede participation; participants reported COVID-19 to be the “new normal” and would not impact their interest or willingness to enroll in the BBS. Most participants reported that transmission mitigation strategies such as the distribution of masks and sanitizer and implementation of social distancing at survey sites may encourage MSM and TGW to participate in the survey. It was reported that ensuring transmission precautions are in place would promote participants to feel safe coming to the survey site and engaging with survey staff. While most participants felt COVID-19 would not impact their willingness to participate, some reported that a worsening epidemic and/or increased government containment measures may impact participation of their peers. Fear of SARS-CoV-2 infection and mobility restrictions were cited as factors that may impede recruitment.

### Impacts of COVID-19 among PWID

Duration of sessions ranged from 80–180 minutes for IDIs and 60–190 minutes for FGDs. PWID IDI participants ranged in age from 18–51 years (Table 3). Most PWID identified as male (82%, Table 3), had received secondary school education (49%), and worked in the informal sector (73%). Of the nine FGDs, four consisted of only males, one consisted of only females, three consisted of males and females, and one consisted of males and TGW. Across towns, the median number of participants in FGDs was 8 (range: 6–9).

**Table 3:** Demographic characteristics among PWID IDI participants, Zambia, 2020.

Characteristic	Lusaka (n = 15, 33%)	Livingstone (n = 15, 33%)	Ndola (n = 15, 33%)	Total (n = 45, 100%)
Median age (range)	24.5 (21–51)	28 (18–41)	34 (23–48)	28 (18–51)
Gender Male Female TGW	14 (93%) 1 (7%) 0 (0%)	11 (73%) 3 (20%) 1 (7%)	12 (80%) 2 (13%) 1 (7%)	37 (82%) 6 (12%) 2 (4%)
Education Primary Secondary Tertiary/vocational	11 (73%) 4 (27%) 0 (0%)	1 (7%) 12 (80%) 2 (13%)	3 (20%) 6 (40%) 6 (40%)	15 (33%) 22 (49%) 8 (18%)
Employment Formal Informal Student Unemployed	1 (7%) 11 (73%) 3 (20%) 0 (0%)	2 (13%) 12 (80%) 1 (7%) 0 (0%)	4 (27%) 10 (67%) 0 (0%) 1 (7%)	7 (16%) 33 (73%) 4 (9%) 1 (2%)
Marital status Single, never married Married Cohabitating Separated/divorced	8 (53%) 2 (13%) 1 (7%) 4 (27%)	12 (80%) 3 (20%) 0 (0%) 0 (0%)	7 (46%) 6 (40%) 0 (0%) 2 (13%)	27 (60%) 11 (24%) 1 (2%) 6 (13%)
Religion Christian Islam None/did not answer	14 (93%) 1 (7%) 0 (0%)	14 (93%) 0 (0%) 1 (7%)	12 (80%) 0 (0%) 3 (20%)	40 (89%) 1 (2%) 4 (9%)

Five primary themes related to the impacts of COVID-19 on PWID emerged from IDIs and FGDs: economic impact, mental health, HIV services, injection practices, and willingness to participate in the upcoming BBS. Illustrative quotes for themes and sub-themes are provided in Table 4.

**Table 4:** Themes, sub-themes, and illustrative quotes on the impacts of the COVID-19 pandemic among PWID, Zambia, 2020.

Sub-theme	Illustrative quote(s)
**Primary theme 1: COVID-19 economic impacts**	
Loss of formal employment	*“I lost my job as [a] lorry boy because they closed the borders and there are only essential trucks that can cross [the border]*. *So*, *it has really done me bad*.*”—Livingstone IDI*, *Male 30 years* *“COVID-19 has had a big impact on me as an individual*, *I could no longer work*.*”—Livingstone IDI*, *Female 27 years*
Loss of informal employment	*“I was not able to move around we were told to stay home and even when I moved*, *we were not allowed to be in groups and if found in groups the police would beat or arrest us*. *So*, *this affected the way I looked for money*.*”—Livingstone IDI*, *Male 22 years* *COVID-19 has affected the respondent’s business as previously before the lockdown she used to sell boxers and pants*. *The closure of borders prevented the respondent from buying and selling pants*. *She then struggled with rentals and was evicted from the house*.*—Livingstone IDI*, *TGW 28 years*
**Primary theme 2: COVID-19 impacts on mental health**	
Stress due to economic impacts of COVID-19	*“My mental health was severely affected when my place of work was closed down under the partial lockdown that mainly affected places like lodges where I was working from*. *After abruptly losing means of having an income*, *I became depressed*.*”—Livingstone IDI*, *Female 27 years* *“The COVID-19 really worried me big time*, *my biggest worry was how were we going to survive*, *how were we going to do business and still make money*.*”—Ndola IDI*, *Male 25 years*
**Primary theme 3: COVID-19 impacts on HIV services**	
Social-economic impact of COVID-19 restrictions as a barrier to accessing HIV services	*“I would go and had no impact on receiving treatment but only transport became an issue coz I became jobless”—Livingstone IDI*, *Male 30 years* *“[I] didn’t access health services because at the hospital [I] would have been required to wear a mask which [I] could not afford*.*”–Lusaka IDI*, *Male 18 years*
Operational changes at health facility such as reduced hours and restrictions on the number of patients indoors	*“I was afraid to contract COVID and they were no drugs at the clinic*, *opening hours were reduced*, *no face mask no service and no transport to the clinic*.*”–Ndola IDI*, *Male 27 years*
Mandatory screening or testing for COVID-19 prior to accessing HIV services	*“When I escorted my friend to the clinic*, *a lot of things were happening there*, *there was a lot of testing and quarantine activities*, *so I ran away because they wanted to detain me*.*”—Ndola*, *Male 25 years*
Avoidance of HIV care seeking due to fear of SARS-CoV-2	*“I would not go to the clinic in fear of getting the COVID-19 virus*, *so I stayed home”—Livingstone IDI*, *Male 33 years* *“Before the coming of COVID-19*, *they used to frequent the clinics for even manageable disease like flu*. *These days it is very tricky to go to the clinics*. *People are very scared of contracting COVID-19 from there*.*”—Lusaka*, *Male 22 years* *“Fear of going to the hospital because of COVID-19 so I didn’t access HIV services from the hospital*.*”—Lusaka*, *Male 51 years*
Ease of access and availability of HIV services	*COVID-19 did not have a negative impact on access to services*. *The participant reported the personnel were as friendly as before though some strict COVID-19 guidelines were put in place*.*–Lusaka IDI*, *Male 22 years* *Participants didn’t really feel the impact because most peer volunteers in Chibolya (township with large concentration of PWID) have [test] kits and condoms in the community kept at their home*.*–Lusaka FGD*, *Males 16+ years*
Non-COVID-19-related barriers to care; stigma experienced at health facilities	*“Junkies have no time to go to the hospital to seek health services because of the stigma from nurses at the hospitals and the long queues at the hospital*.*”—Livingstone IDI*, *Male 18 years* *“I am no fan of clinics*, *I don’t go [to] such places*. *There is too much stigma and no confidentiality*.*”—Ndola IDI*, *Male 48 years*
**Primary theme 4: COVID-19 impacts on injection practices**	
Increased needle and syringe sharing due to their availability and cost	*“There were no needles available*, *so we were forced to start sharing [needles and] syringes more often than before”–Livingstone IDI*, *Male 22 years* *“There was an impact as we had to share the few needles and syringes available*. *This sharing made the whole practice unsafe*, *but we had no choice because of the addiction*.*”—Livingstone IDI*, *Female 27 years* *“My safety of injection has not been affected that much*, *maybe in terms of health where we now inject in groups of 20 so that you afford materials*. *This exposes us to HIV/AIDS*.*”—Ndola IDI*, *Male 25 years* *“Reduced safety because more people engaged in what is known as Bluetooth which means exchanging blood and sharing needles used when the money to purchase the drug was put (together) contributed for*. *Also*, *many people reused needles even if they are single use needles*.*”—Lusaka FGD*, *Males 16+ years*
Increased multiple re-use of needles and syringes	*“It [COVID-19 restrictions] put us in danger*, *spoiled our lives*. *We didn’t have access to clean needles*, *so we ended up re-using and sharing the old needles that we have*. *Sometimes if available we would put the syringe/needle into spirit*. *But in most cases five of us would use one syringe*. *After use we will keep it for another time to use*, *we will not throw it away*.*”—Ndola FGD*, *Males <40 years* *The Safety has been compromised as the respondent no longer has access to the needles and syringes and has resorted to boiling water and cleaning the needles with hot water and a cloth*.*—Livingstone IDI*, *TGW 28 years*
Reduced frequency of injecting due to limited drug supply	*“Because of no money the supplier left the country*. *So*, *because of this my supply has reduced and reduced my daily injecting from three times a day to twice a day*. *Some reduced from daily to once a week*.*”–Ndola FGD*, *Males 40+ years* *“Since all borders were closed during lockdown*, *a lot of PWID got sick due to withdrawal symptoms*. *Very few suppliers hence limited injecting drugs*.*”–Lusaka FGD*, *Males and Females 25+ years* *“Yes*, *I was affected a lot because I had to reduce on the number of times I injected drugs due to low supply of the drug*. *We even started sharing needles because we were not allowed to move around*, *and certain shops were closed*.*”—Livingstone IDI*, *Male 29 years*
**Primary theme 5: Willingness to participate in upcoming BBS in the context of COVID-19**	
COVID-19 is unlikely to impede participation as COVID-19 is the “new normal”	*“COVID-19 will not affect the upcoming survey as most people have learnt to live [with] the new normal*.*”–Livingstone FGD*, *Males and TGW 25+ years* *“Most of my friends will be willing to participate in this survey because it focuses on their health*. *COVID-19 will not scare them or affect their willingness to join this survey*.*”—Ndola IDI*, *Male 23 years*
Transmission mitigation strategies such as distribution of masks and sanitizer and implementation of social distancing may encourage participation	*“The COVID-19 has affected a lot of things*, *but it cannot stop my peers and I to participate in the upcoming survey since with the passing of the new statutory instrument we are living in the new normal*. *We will wear masks and come*.*”—Ndola IDI*, *Male 27 years* *“We will not be affected in any way as long as we mask up we can all take part”—Livingstone IDI*, *Male 33 years*

COVID-19 restrictions and border closures led to unemployment and loss of income for many participants, as well as shortages and increased price of drugs, needles, and syringes, which contributed to emotional distress. Participants across towns and data collection methods reported increased needle-sharing and re-use of needles due to COVID-19 economic effects and limited drug supply. Some participants also reported reduced frequency of drug injection due to limited drug supply.

Participants reported not accessing HIV services due to COVID-19-related fears, long queues, limited operating hours, reassignment of healthcare workers, crowded conditions, and mask and COVID-19 testing requirements. Chronic issues for accessing services irrespective of the pandemic were also reported, including stigma and discrimination by healthcare workers and transportation costs. Others reported accessing HIV services, including pre-exposure prophylaxis (PrEP), with no difficulty.

Like MSM/TGW, PWID were willing to participate in the upcoming BBS despite the COVID-19 pandemic; participants echoed that COVID-19 was the “new normal” and indicated it would have little impact on their willingness to participate in the survey. Participants were motivated to engage in the BBS as they felt it was important for their health. Many participants described the importance of adhering to transmission mitigation strategies at the survey site, including wearing masks and use of hand sanitizer.

## Discussion

Findings from this formative assessment highlight areas in which COVID-19 and government restrictions in response to COVID-19 have impacted KP in Zambia, including impacts on sexual risk behaviors, safe injection practices, and access to HIV services, and can inform both KP programming as well as surveillance efforts among KP in the context of COVID-19 and/or other emerging pandemic threats. Importantly, while the focus of this analysis was primarily to explore COVID-19 impacts, results also elucidate structural barriers to HIV service delivery for KP irrespective of COVID-19 such as stigma and discrimination and violence. Data on KP in Zambia are sparse likely due to the punitive and non-protective legal and social context, and this assessment provides evidence of areas where improvements are needed to align programs with UNAIDS 2025 10-10-10 targets for societal enablers. To remove social and legal impediments towards an enabling environment these targets aim to ensure less than 10% of countries have punitive legal and policy environments that deny access to justice, less than 10% of KP and people living with HIV experience stigma and discrimination, and less than 10% of women, girls, people living with HIV and KP experience gender inequality and violence [20].

Reduced access to HIV prevention, care, and treatment services among KP during COVID-19 has been documented in other settings globally, including in other low- and middle-income countries [21–23]. Like findings elsewhere [21–23], KP in this formative assessment experienced barriers accessing HIV services, including HIV testing, as well as limited supply of condoms and lubricants and drug stockouts at the health facility. Both MSM/TGW and PWID described barriers related to health facility operational changes and COVID-19 requirements as well as avoidance of HIV care seeking due to fear of potential SARS-CoV-2 exposure at the health facility. As the COVID-19 epidemic evolves in Zambia or future pandemic threats are posed, scale-up of community-based work and improvements in supply chain may be important to ensure continued coverage of HIV services during a public health emergency.

COVID-19 has also disrupted illicit supply chains. These disruptions have impacted drug supply, access to safe injection equipment, and drug injection practices for PWID in some countries [24]. In Zambia, loss of income and lack of affordable needles led to an increase in unsafe needle use and sharing practices, highlighting a need for harm reduction strategies and PWID-tailored services. Few organizations provide services specifically for PWID in Zambia, and those that do are limited in their ability to provide comprehensive harm reduction services [9]. Ensuring an enabling environment for organizations to provide harm reduction services for PWID, particularly in the context of COVID-19 or other emerging pandemic threats, warrant prioritization. Additionally, human rights-based training approaches for healthcare workers may reduce stigmatization at the health facility, a chronic barrier to care described by our participants.

MSM/TGW participants highlighted the implementation of innovations in response to COVID-19; KP organizations may consider scaling up these types of innovations, including HIV self-testing, mobile clinics, and appointment reminders, as well as scaling up or strengthening other innovative delivery approaches such as multi-month dispensing of PrEP and ART, community ART groups, and other differentiated service delivery models, telehealth support across the cascade of services, and mailed testing and prevention commodities [25]. Moreover, as MSM/TGW in this formative assessment adapted to meet sexual partners/clients via social media in response to COVID-19 restrictions, KP organizations may utilize similar platforms to engage MSM/TGW and promote HIV services in the context of COVID-19 or other emerging pandemic threats; this may be appropriate given the legal context and can reduce risk of exposure.

In this formative assessment, COVID-19 had little impact on KP’s willingness to participate in the upcoming BBS. While findings that KP can successfully be recruited for BBS participation are promising, considerations for implementing BBS among KP in the context of COVID-19 or future pandemic threats must extend beyond willingness to participate. Adherence to transmission mitigation strategies including social distancing and mask wearing is important as emphasized by participants in this study; BBS methods may warrant adaptations including use of audio computer-assisted self-interview, limits in the number of KP visiting data collection sites, use of appointments in lieu of walk-ins, routine testing of BBS staff, and/or outdoor interview administration to minimize potential for transmission, among others. Moreover, as the COVID-19 epidemic or future epidemic threats evolve, severity of restrictions during BBS data collection may fluctuate which may impact participants’ willingness to participate, their ability to recruit, and/or their self-reported personal network sizes. As network size is used to generate weights for BBS using RDS, weighting approaches for RDS surveys conducted in the context of COVID-19 or future pandemic threats may require adjustment. Additionally, as described in this assessment, COVID-19 had impacts on access to HIV services, sexual behavior, and injection practices among KP and BBS estimates should be interpreted with this considered.

The primary limitation of this study is that data were not audio recorded or transcribed due to local ethical concerns related to participant confidentiality. We sought to overcome this limitation by providing a five-day intensive didactic training on qualitative research methods to all formative assessment staff and assigning two trained interviewers to document IDIs/FGDs sessions including observations and verbatim participant quotes. Moreover, while this is a limitation, the absence of transcription in response to confidentiality concerns have been documented in other formative assessments in similar contexts [15], and results remain important contributions to the literature nonetheless. Moreover, participants in this formative assessment were recruited with support from KP community mobilizers and civil society organizations, and results represent perspectives from individuals who were purposively recruited. Despite limitations, we feel these results are necessary to inform programming for and surveillance of MSM/TGW and PWID in Zambia, where little information on these KP have been published.

Taken together, findings elucidate COVID-19’s impacts on sexual risk behaviors, safe injection practices, access to HIV services, and willingness to participate in BBS for these groups, and have important programming implications. KP overall reported experiencing barriers accessing HIV services due to COVID-19 as well as structural barriers accessing care. MSM/TGW reported limited supply of condoms and lubricants at health facilities which led to increased engagements in condomless sex, and PWID attributed unsafe needle use and sharing to loss of income and lack of affordable needles during pandemic-related restrictions. Participants also noted innovations to address barriers to care in response to COVID-19. To maintain gains in the HIV response in this context and in the context of future pandemic threats, strengthening harm reduction strategies and improvements in access to HIV services, through scale-up of innovations, are and will continue to be necessary.

## Supporting information

S1 Questionnaire (DOCX)
